# Qu­anti­tative analysis of weak non-covalent inter­actions in (*Z*)-3-(4-chloro­phen­yl)-2-phenyl­acrylo­nitrile: insights from *PIXEL* and Hirshfeld surface analysis

**DOI:** 10.1107/S2056989019003694

**Published:** 2019-03-26

**Authors:** Mani Udayakumar, Margarita Cerón, Paulina Ceballos, Judith Percino, Subbiah Thamotharan

**Affiliations:** a Biomolecular Crystallography Laboratory, Department of Bioinformatics, School of Chemical and Biotechnology, SASTRA Deemed University, Thanjavur 613 401, India; b Unidad de Polímeros y Electrónica Orgánica, Instituto de Ciencias, Benemérita Universidad Autónoma de Puebla, Val3-Ecocampus Valsequillo, Independencia O2 Sur 50, San Pedro Zacachimalpa, Puebla, CP 72960, Mexico

**Keywords:** crystal structure, acrylo­nitrile, conjugation, hydrogen bonding, C—H⋯π inter­actions, PIXEL, Hirshfeld surface, two-dimensional fingerprint plots.

## Abstract

In the solid state, the title compound, C_15_H_10_ClN, is disordered over two orientations with a refined occupancy ratio of 0.86 (2):0.14 (2). The crystal structure is mainly stabilized by inter­molecular C—H⋯N and C—H⋯Cl hydrogen bonds, and C—H⋯π inter­actions. The mol­ecules pack in columns and adjacent columns are linked by weak C—H⋯Cl inter­actions. The *PIXEL* energy analysis suggests that the inter­molecular C—H⋯π inter­actions form a strong dimer in the major component. Hirshfeld analysis reveals that H⋯C, H⋯H, H⋯Cl and H⋯N contacts are the most important contributors to the crystal packing.

## Abstract 2

The crystal and mol­ecular structures of (*Z*)-3-(4-chloro­phen­yl)-2-phenyl­acrylo­nitrile are reported and the weak non-covalent inter­actions present in the crystal structure have been investigated.

## Chemical context   

Acrylo­nitrile compounds have been used as building blocks in flavonoid pigments (Fringuelli *et al.*, 1994 ▸) and anti­cancer agents (Özen *et al.*, 2016 ▸). Some of these derivatives have been used to produce light-emitting diodes (LEDs) (Maruyama *et al.*, 1998 ▸; Segura *et al.*, 1999 ▸). Owing to the versatile physicochemical and biological properties of acrylo­nitrile derivatives, we have been investigating the optical properties of several (*Z*)-3-(substituted phenyl)-2-(pyrid­yl)acrylo­nitrile compounds with different donor and acceptor moieties (Percino *et al.*, 2010 ▸, 2011 ▸, 2014*a* ▸,*b* ▸, 2016*a* ▸,*b* ▸, 2017 ▸). Recently, we explored various (*Z*)-3-(4-halophen­yl)-2-(pyridin-2/3/4-yl)acrylo­nitrile derivatives in order to understand the role of halogen substituents in the context of optical properties and supra­molecular associations in the solid state (Venkatesan *et al.*, 2018 ▸).
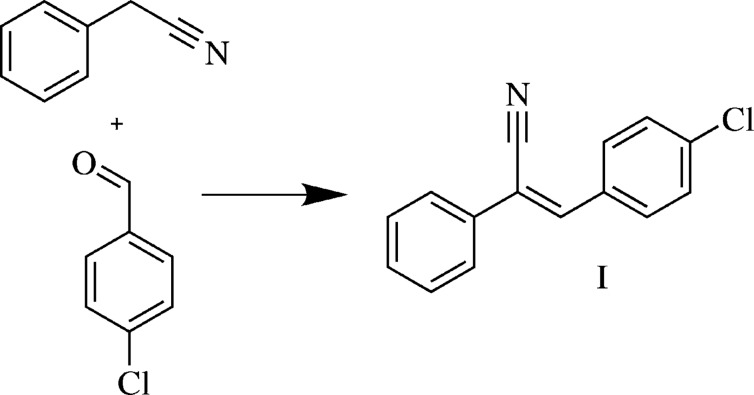


In this work, we report the synthesis and the crystal and mol­ecular structures of an acrylo­nitrile derivative, namely (*Z*)-3-(4-chloro­phen­yl)-2-phenyl­acrylo­nitrile (**I**). We also report herein a detailed analysis of the inter­molecular inter­actions for different mol­ecular pairs observed in **I** using the *PIXEL* method (Gavezzotti, 2002 ▸, 2011 ▸). Hirshfeld surface analysis (Spackman & Jayatilaka, 2009 ▸) was also performed to visualize the short contacts in the crystal of **I** and to determine the relative contributions of the various non-covalent inter­actions present in the crystal structure using two-dimensional (2D) fingerprint plots (Spackman & McKinnon, 2002 ▸; McKinnon *et al.*, 2007 ▸). We also highlight the importance of the weak halogen bonds observed in the crystal structure.

## Computational details   

Structural optimization was carried out using *GAUSSIAN09* (Frisch *et al.*, 2013 ▸) with the M06-2X/cc-pVTZ level of theory followed by vibrational frequency calculations. The lattice and inter­molecular inter­action energies were calculated using the *CLP-PIXEL* program (Version 3.0; Gavezzotti, 2002 ▸, 2011 ▸). For the inter­molecular inter­action energy calculations, the crystal structure geometry along with normalized C—H bond lengths to their respective neutron values (Allen, 1986 ▸) was used and the electron density has been obtained at the MP2/6-31G(d,p) level of theory using *GAUSSIAN09*.

## Structural commentary   

**Figure 1:**
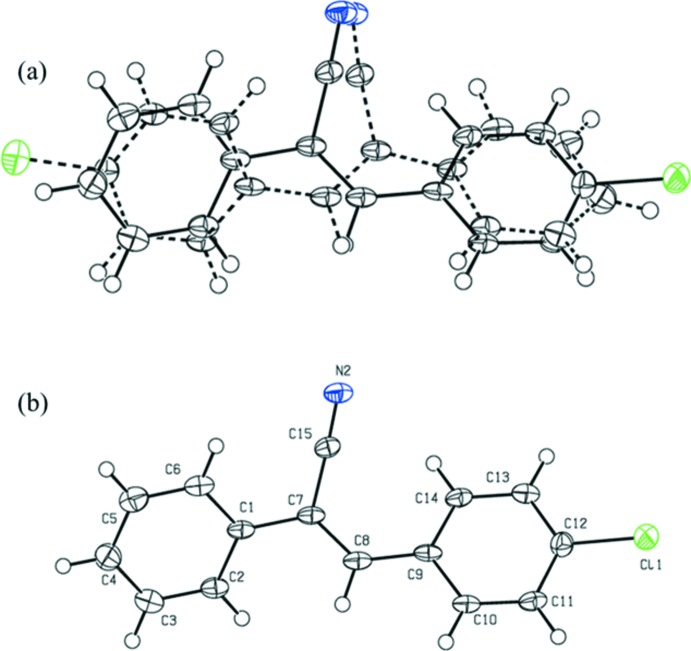
(*a*) The disordered components of compound **I** (major shown with solid lines and minor with broken lines) and (*b*) displacement ellipsoids of the major disordered component of **I** at the 50% pobability level, with the atom-labelling scheme.

The mol­ecular structure of compound **I** is shown in Fig. 1 ▸. The whole mol­ecule is disordered over two orientations with a refined occupancy ratio of 0.86 (2):0.14 (2). Only the major component is considered for further analysis and discussion. The bond lengths in **I** clearly indicate the presence of electron delocalization throughout the mol­ecule. The geometrical features of the mol­ecule were further analyzed using the *MOGUL* geometry check utility available in *Mercury* (Macrae *et al.*, 2008 ▸). The result suggests that the torsion angles C8—C7—C15—N2 [−166.6 (2)°] and C1—C7—C15—N2 [10.5 (2)°] are unusual. The mol­ecule adopts a twisted conformation and the dihedral angle between the planes of the phenyl (C1–C6) and 4-chloro­phenyl (C9–C14) rings is 51.91 (8)°. When the unsubstituted phenyl ring in **I** was replaced by a pyridine ring (Venkatesan *et al.*, 2018 ▸), the mol­ecular twist was reduced by at least 50%, and in pyridine containing compounds, the dihedral angles between the two rings are in a range of *ca* 1–27° (Cambridge Structural Database; Groom *et al.*, 2016 ▸).

**Figure 2:**
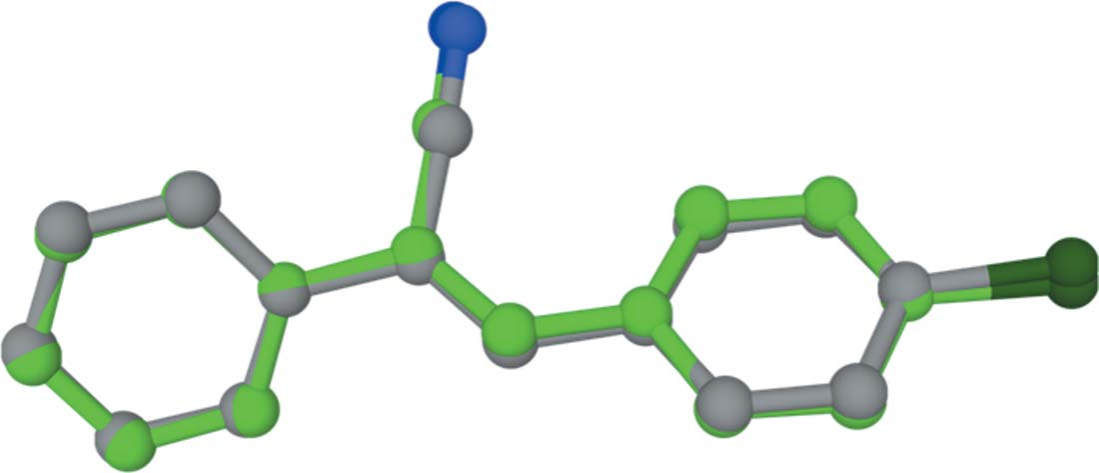
Structural overlay of the X-ray (grey) and optimized (green) structures.

To understand the conformational flexibility of **I**, we performed a structural optimization using the *GAUSSIAN09* program (Frisch *et al.*, 2013 ▸), without any constraints. The vibrational frequency calculation confirmed that the optimized structure is found to be the true energy minima on the potential energy surface, since there were no negative frequencies observed for the optimized geometries. The X-ray and optimized structures superimpose well, with an r.m.s. deviation of 0.13 Å (Fig. 2 ▸).

## Supra­molecular features   

**Figure 3:**
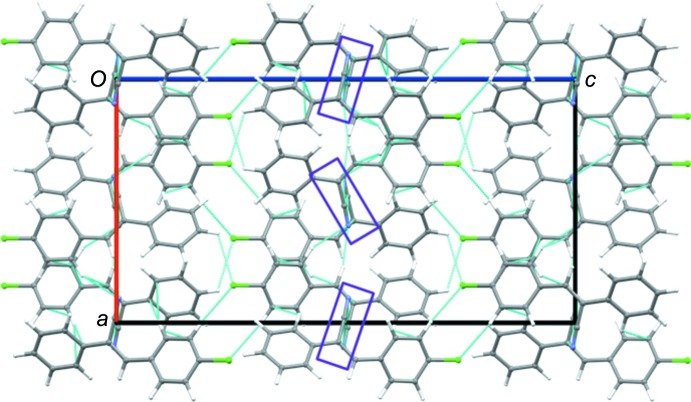
A view along the *b* axis of the crystal packing of compound **I**, showing the nitrile stacking in the purple rectangles.

In the crystal, mol­ecules are arranged in a columnar packing mode *via* inter­molecular C—H⋯π, C—H⋯N and C—H⋯Cl inter­actions (Table 1 ▸ and Fig. 3 ▸). Adjacent columns are inter­connected by halogen bonds (C—H⋯Cl). Within the column, there is nitrile–nitrile stacking and mol­ecules are inter­linked by C—H⋯π and C—H⋯N inter­actions (Table 1 ▸).

## Lattice and inter­molecular inter­action energies   

The lattice energy calculations reveal that the crystal packing is predominantly stabilized through dispersion energy (71%) and the electrostatic (Coulombic + polarization) energy contributes 29% towards the stabilization of the crystal structure. The total lattice energy (−28.9 kcal mol^−1^) is the sum of the Coulombic (−10.5 kcal mol^−1^), polarization (−4.7 kcal mol^−1^), dispersion (−36.6 kcal mol^−1^) and repulsion (22.9 kcal mol^−1^) terms. Furthermore, different motifs formed in the major component of **I** and their energetics are discussed below (Table 2 ▸).

**Figure 4:**
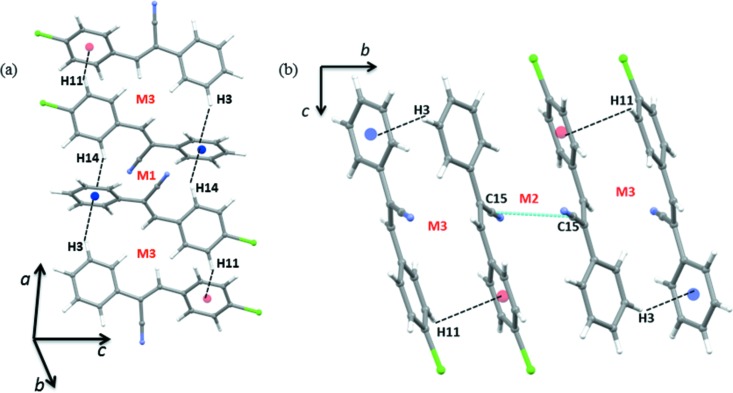
(*a*) The mol­ecular chain generated by inter­molecular C—H⋯π inter­actions (motif sequence M3⋯M1⋯M3) and (*b*) adjacent M3 motifs inter­linked by nitrile–nitrile stacking (motif M2). The centroids are shown as small spheres (*Cg*1 blue and *Cg*2 red).

Inversion-related mol­ecules form the strongest dimer (motif M1) which is held by inter­molecular C—H⋯π inter­actions with an inter­action energy of −9.5 kcal mol^−1^. As expected, the dispersion contribution (70%) is more significant towards the stabilization of this dimer. Further, this dimer is flanked on both sides by other mol­ecules. As shown in Fig. 4 ▸(*a*), these mol­ecules inter­act with the central dimer (motif M1) through two C—H⋯π inter­actions (motif M3; inter­action energy = −7.3 kcal mol^−1^). It is to be noted that the motif M3 is more dispersive in nature (78%) than motif M1. The nitrile group of one mol­ecule stacks with the nitrile group of an inversion-related mol­ecule (motif M2; inter­action energy = −8.7 kcal mol^−1^ and 71% dispersion contribution). The shortest distance observed between two C15 atoms is 3.274 (4) Å and the motif M2 is also flanked on both sides by motif M3. These motifs act together to link the mol­ecules into a chain which runs parallel to the *b* axis (Fig. 4 ▸ *b*).

**Figure 5:**
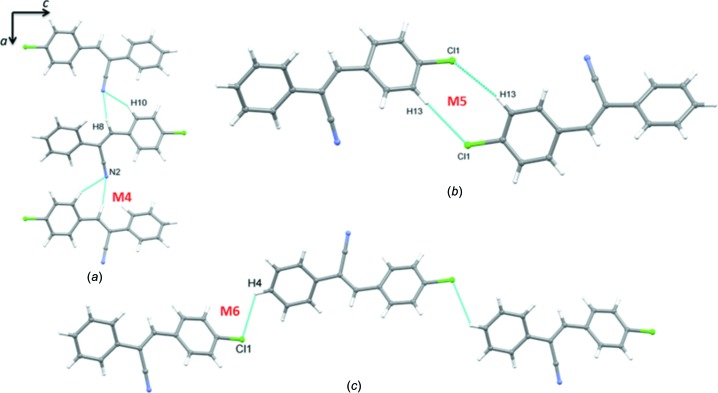
(*a*) The mol­ecular chain formed by three-centred C—H⋯N inter­actions, (*b*) a closed mol­ecular dimer generated by inter­molecular C—H⋯Cl inter­actions and (*c*) a *C*(12) chain formed by inter­molecular C—H⋯Cl inter­actions.

Motif M4 (inter­action energy = −5.9 kcal mol^−1^) is stabilized by three-centred inter­molecular C—H⋯N inter­actions in which the nitrile N atom acts as an acceptor and the vinylic proton (H9) and one of the protons (H10) of chloro­phenyl ring are involved as donors (Fig. 5 ▸). These three-centred inter­actions link the mol­ecules into a chain which runs parallel to the *a* axis. 53% of the electrostatic and 47% of the dispersion energy contribute towards stabilization of motif M4.

**Figure 6:**
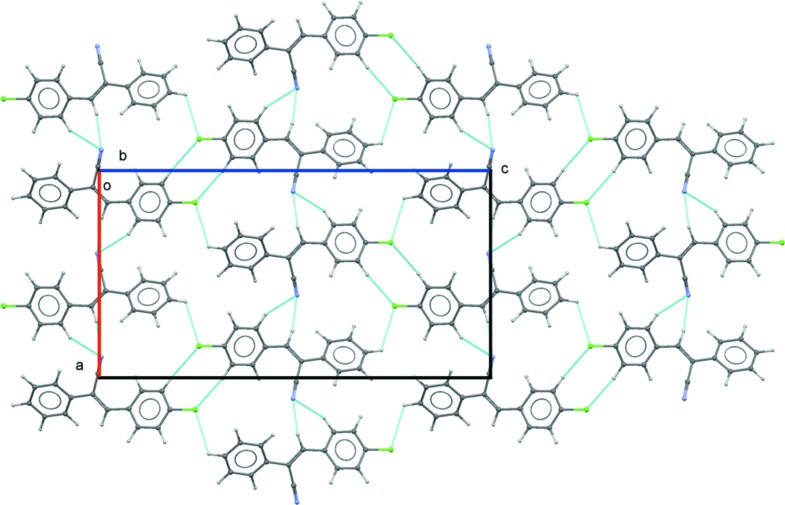
The mol­ecular sheet assembled by inter­molecular C—H⋯N and C—H⋯Cl inter­actions.

The energetically least-stable dimers (motifs M5 and M6) are formed by inter­molecular C—H⋯Cl inter­actions (Fig. 5 ▸). These two inter­actions help to link adjacent columns in the crystal, as mentioned above. The mol­ecules form an 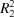
(8) loop in the case of motif M5, with an inter­action energy of −2.8 kcal mol^−1^. We note that the dispersion energy (67%) contributes nearly double that of the electrostatic energy (33%) for the stabilization of this motif. Further, a mol­ecular chain is related to motif M6 (inter­action energy = −1.6 kcal mol^−1^) propagating along the *c* axis direction. This dimer is more dispersive in nature and 75% of the dispersion energy contributes towards the stabilization. Motifs M4–M6 combine to form sheets parallel to the *ac* plane (Fig. 6 ▸).

## Hirshfeld surface analysis and 2D fingerprint plots   

**Figure 7:**
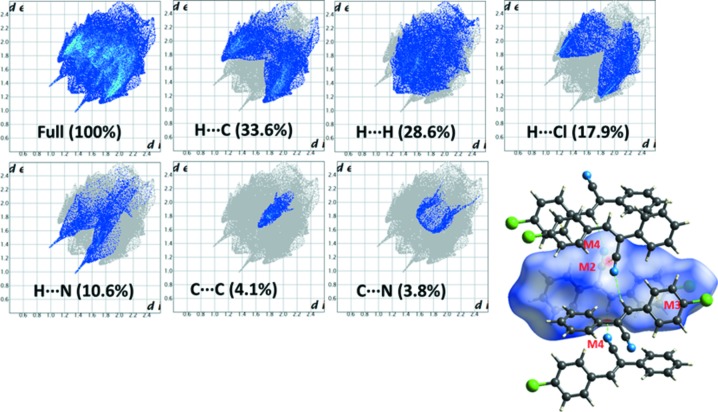
2D fingerprint plots for different inter­molecular contacts and the Hirshfeld surface mapped over *d*_norm_ to hightlight the short inter­molecular contacts for the major disordered component of **I**.

The Hirshfeld surface analysis (Spackman & Jayatilaka, 2009 ▸) and the associated 2D fingerprint plots (McKinnon *et al.*, 2007 ▸) were performed with *CrystalExplorer17* (Turner *et al.*, 2017 ▸) for both the major and the minor disordered components. For each component, the occupancies of all atoms were made equal to 1. Hirshfeld surface (HS) analysis was carried out in order to gain more insight into the nature and extent of the inter­molecular inter­actions and to qu­antify the relative contributions of the different non-covalent inter­actions that exist in the crystal. The HS surface was mapped over *d*_norm_ and the diagram reveals that motifs M2 and M4 are visible as red spots on the HS (Fig. 7 ▸) in the major disordered component. It is to be noted that a pale-red spot is noticed for motif M3 when compared to the other two motifs. As mentioned above, motif M4 has two inter­molecular C—H⋯N inter­actions and one of them is found to be a close contact (C8—H8⋯N2).

2D fingerprint plots for the major and the minor components are illustrated in Figs. 7 ▸ and 8 ▸. For the major component of **I**, it is found that the contributions for the H⋯C (33.6%) and H⋯H (28.6%) contacts are relatively high in comparison to other non-covalent inter­actions (Fig. 7 ▸). It is of inter­est to note that the H⋯Cl contacts also contribute substanti­ally (17.9%) to the crystal packing. As noted above, neighbouring columns are inter­linked in the crystal *via* inter­molecular H⋯Cl contacts. The inter­molecular H⋯N contacts contribute 10.6% towards the crystal packing. The other contacts, such as C⋯C (4.1%) and C⋯N (3.8%), also supplement the overall crystal packing. The former contact represents the motifs M2 and M3, while the latter contact is mainly due to the stacking of the nitrile groups.

**Figure 8:**
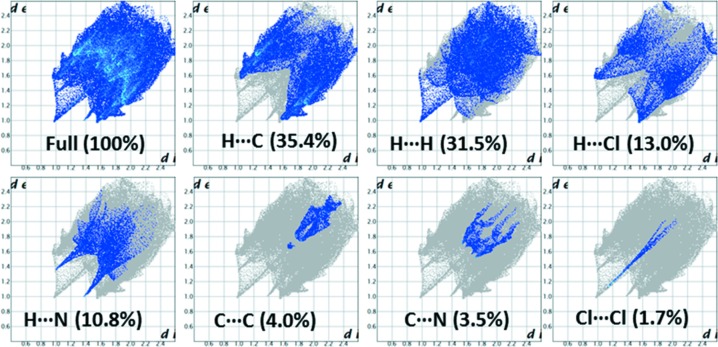
2D fingerprint plots for the different inter­molecular contacts and their relative contributions for the minor disordered component of **I**.

In the case of the minor component, the relative contributions of some of the inter­molecular contacts are very similar to those for the major component, as shown in Fig. 8 ▸. However, the H⋯Cl contacts are reduced by 4.9%. This difference clearly indicates the importance of halogen inter­actions in the major component of the title compound.

## Database survey   

A search of the Cambridge Structural Database (CSD, Version 5.40, update of February 2019; Groom *et al.*, 2016 ▸) using the (*Z*)-2,3-di­phenyl­acrylo­nitrile skeleton yielded 306 hits, which include multiple reports of a number of structures. Limiting the search to structures with a halogen atom attached to the phenyl ring, as in the title compound, yielded 13 hits. Two structures are similar to the title compound, namely 2-(4-amino­phen­yl)-3-(4-bromo­phen­yl)acrylo­nitrile (CSD refcode IYIBOJ; Bai *et al.*, 2016 ▸) and (*Z*)-3-(2-chloro-6-fluoro­phen­yl)-2-(4-meth­oxy­phen­yl)acrylo­nitrile (KEVQOS; Naveen *et al.*, 2006 ▸). Here the planes of the aryl rings are inclined to each other by 66.16 (13)° in IYIBOJ and 57.43 (19)° in KEVQOS. In **I**, this dihedral angle is 51.91 (8)° in the major disordered component and 61.8 (13)° in the minor disordered component.

## Synthesis and crystallization   

A mixture of phenyl­aceto­nitrile (0.53 ml, 4.6 mmol) and 4-chloro­benzaldehyde (4.6 mmol, 0.65 g) was stirred at room temperature for 10 min. Subsequently, the temperature was increased gradually to 403 K and maintained at that tem­per­ature for 39 h. Initially, the mixture was colourless and then became viscous and dark. This viscous solution was cooled, treated with hexane and finally filtered. The filtrate contained small colourless crystals. Further purification of the title compound (yield 83%, m.p. 368–370 K) was carried out by recrystallization from hexane. Colourless plate-like crystals, suitable for X-ray diffraction analysis, were obtained by slow evaporation of a solution of **I** in ethanol at 277 K after a period of 7 d.

## Refinement   

Crystal data, data collection and structure refinement details are summarized in Table 3 ▸. The whole mol­ecule was disordered and the major and minor components of the disorder refined to 0.86 (2) and 0.14 (2), respectively. All H atoms were placed in calculated positions and treated as riding, with C—H = 0.95 Å and *U*_iso_(H) = 1.2*U*_eq_(C).

## Supplementary Material

Crystal structure: contains datablock(s) I, Global. DOI: 10.1107/S2056989019003694/su5483sup1.cif

Structure factors: contains datablock(s) I. DOI: 10.1107/S2056989019003694/su5483Isup2.hkl

CCDC reference: 1903772

Additional supporting information:  crystallographic information; 3D view; checkCIF report

## Figures and Tables

**Table 1:** Hydrogen-bond geometry (Å, °) *Cg*1 and *Cg*2 are the centroids of rings C1–C6 and C9–C14 of the major disordered component. *Cg*1′ and *Cg*2′ are the centroids of rings C1′–C6′ and C9′–C14′ of the minor disordered component.

*D*—H⋯*A*	*D*—H	H⋯*A*	*D*⋯*A*	*D*—H⋯*A*
C5—H5⋯Cl1′^i^	0.95	2.69	3.292 (5)	122
C8—H8⋯N2^ii^	0.95	2.46	3.361 (6)	157
C8—H8⋯N2′^ii^	0.95	2.53	3.44 (4)	160
C14—H14⋯N2′	0.95	2.54	3.22 (6)	129
C3—H3⋯*Cg*1^iii^	0.95	2.99	3.860 (4)	153
C3—H3⋯*Cg*2′^iii^	0.95	2.95	3.784 (9)	148
C11—H11⋯*Cg*2^iv^	0.95	2.96	3.418 (7)	111
C11—H11⋯*Cg*1′^iv^	0.95	2.97	3.486 (15)	115
C14—H14⋯*Cg*1^v^	0.95	2.81	3.503 (3)	130
C14—H14⋯*Cg*2′^v^	0.95	2.84	3.585 (8)	136
C3′—H3′⋯*Cg*2^iv^	0.95	2.59	3.32 (6)	134
C3′—H3′⋯*Cg*1′^iv^	0.95	2.62	3.39 (6)	139
C6′—H6′⋯*Cg*1^v^	0.95	2.85	3.52 (2)	129
C6′—H6′⋯*Cg*2′^v^	0.95	2.93	3.64 (2)	132

Symmetry codes: (i) 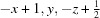; (ii) 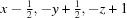; (iii) 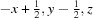; (iv) 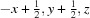; (v) 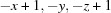.

**Table 2:** Inter­molecular inter­action energies (in kcal mol^−1^) for different mol­ecular pairs observed in the major component of the title compound; CD is the centroid-to-centroid distance

Motif	CD (Å)	Symmetry	*E*_Coul_	*E*_pol_	*E*_energy-dispersive_	*E*_rep_	*E*_tot_	Possible inter­actions	Geometry (Å, °)^*a*^
M1	5.163	−*x* + 1, −*y*, −*z* + 1	−4.0	−1.4	−12.5	8.3	−9.5	C14—H14⋯*Cg*1	2.81, 130
M2	4.820	−*x* + 1, −*y* + 1, −*z* + 1	−3.1	−1.5	−11.5	7.4	−8.7	C15⋯C15(π–π)	3.274 (4)
M3	5.122	−*x* + 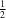 , *y* − 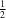 , *z*	−1.9	−1.0	−10.0	5.6	−7.3	C3—H3⋯*Cg*1	2.99, 153
		−*x* + 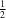 , *y* + 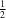 , *z*						C11—H11⋯*Cg*2	2.96, 111
M4	6.925	*x* − 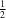 , −*y* + 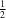 , −*z* + 1	−4.3	−1.6	−5.2	5.3	−5.9	C8—H8⋯N2	2.34, 156
								C10—H10⋯N2	2.66, 143
M5	11.134	−*x* + 1, *y*, −*z* + 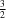	−1.1	−0.5	−3.2	1.8	−2.8	C13—H13⋯Cl1	2.95, 152
M6	13.104	−*x* + 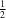 , −*y* + 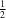 , *z* − 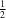	−0.6	−0.4	−2.8	2.1	−1.6	C4—H4⋯Cl1	2.98, 114

Note: (*a*) neutron values are given for all *D*—H⋯*A* inter­actions.

**Table 3:** Experimental details

Crystal data	
Chemical formula	C_15_H_10_ClN
*M*_r_	239.69
Crystal system, space group	Orthorhombic, *P* *b* *c* *n*
Temperature (K)	100
*a*, *b*, *c* (Å)	13.3417 (8), 7.1030 (5), 25.1418 (18)
*V* (Å^3^)	2382.6 (3)
*Z*	8
Radiation type	Cu *K*α
μ (mm^−1^)	2.61
Crystal size (mm)	0.31 × 0.29 × 0.05

Data collection	
Diffractometer	SuperNova, Dual, Cu at zero, Atlas
Absorption correction	Analytical (*CrysAlis PRO*; Agilent, 2012 ▸)
*T*_min_, *T*_max_	0.560, 0.889
No. of measured, independent and observed [*I* > 2σ(*I*)] reflections	6681, 2132, 1930
*R*_int_	0.031
(sin θ/λ)_max_ (Å^−1^)	0.598

Refinement	
*R*[*F* ^2^ > 2σ(*F* ^2^)], *wR*(*F* ^2^), *S*	0.050, 0.122, 1.10
No. of reflections	2132
No. of parameters	212
No. of restraints	44
H-atom treatment	H-atom parameters constrained
Δρ_max_, Δρ_min_ (e Å^−3^)	0.28, −0.20

Computer programs: *CrysAlis PRO* (Agilent, 2012 ▸), *SHELXS2013* (Sheldrick, 2008 ▸), *SHELXL2014* (Sheldrick, 2015 ▸), *PLATON* (Spek, 2009 ▸), *Mercury* (Macrae *et al.*, 2008 ▸) and *publCIF* (Westrip, 2010 ▸).

## supplementary crystallographic information

### Crystal data

**Table:**

C_15_H_10_ClN	*D*_x_ = 1.336 Mg m^−^^3^
*M**_r_* = 239.69	Cu *K*α radiation, λ = 1.54178 Å
Orthorhombic, *P**b**c**n*	Cell parameters from 3085 reflections
*a* = 13.3417 (8) Å	θ = 4.8–67.4°
*b* = 7.1030 (5) Å	µ = 2.61 mm^−^^1^
*c* = 25.1418 (18) Å	*T* = 100 K
*V* = 2382.6 (3) Å^3^	Plate, colourless
*Z* = 8	0.31 × 0.29 × 0.05 mm
*F*(000) = 992	

### Data collection

**Table:**

SuperNova, Dual, Cu at zero, Atlas diffractometer	1930 reflections with *I* > 2σ(*I*)
Radiation source: SuperNova (Cu) X-ray Source	*R*_int_ = 0.031
ω scans	θ_max_ = 67.3°, θ_min_ = 3.5°
Absorption correction: analytical (CrysAlisPro; Agilent, 2012)	*h* = −15→11
*T*_min_ = 0.560, *T*_max_ = 0.889	*k* = −6→8
6681 measured reflections	*l* = −23→30
2132 independent reflections	

### Refinement

**Table:**

Refinement on *F*^2^	Primary atom site location: structure-invariant direct methods
Least-squares matrix: full	Secondary atom site location: difference Fourier map
*R*[*F*^2^ > 2σ(*F*^2^)] = 0.050	Hydrogen site location: inferred from neighbouring sites
*wR*(*F*^2^) = 0.122	H-atom parameters constrained
*S* = 1.10	*w* = 1/[σ^2^(*F*_o_^2^) + (0.0441*P*)^2^ + 2.5669*P*] where *P* = (*F*_o_^2^ + 2*F*_c_^2^)/3
2132 reflections	(Δ/σ)_max_ = 0.001
212 parameters	Δρ_max_ = 0.28 e Å^−^^3^
44 restraints	Δρ_min_ = −0.20 e Å^−^^3^

### Special details

**Table:**

Geometry. All esds (except the esd in the dihedral angle between two l.s. planes) are estimated using the full covariance matrix. The cell esds are taken into account individually in the estimation of esds in distances, angles and torsion angles; correlations between esds in cell parameters are only used when they are defined by crystal symmetry. An approximate (isotropic) treatment of cell esds is used for estimating esds involving l.s. planes.

### Fractional atomic coordinates and isotropic or equivalent isotropic displacement parameters (Å^2^)

**Table:**

	*x*	*y*	*z*	*U*_iso_*/*U*_eq_	Occ. (<1)
Cl1	0.34667 (5)	0.49268 (11)	0.74493 (3)	0.0369 (2)	0.8598 (17)
C12	0.3468 (2)	0.4040 (5)	0.68021 (11)	0.0258 (7)	0.8598 (17)
C11	0.2585 (4)	0.4067 (10)	0.65042 (19)	0.0251 (12)	0.8598 (17)
H11	0.1975	0.4506	0.6654	0.030*	0.8598 (17)
C10	0.26234 (19)	0.3441 (7)	0.59886 (15)	0.0230 (8)	0.8598 (17)
H10	0.2031	0.3475	0.5779	0.028*	0.8598 (17)
C9	0.35071 (18)	0.2756 (4)	0.57609 (11)	0.0211 (6)	0.8598 (17)
C14	0.43690 (19)	0.2651 (4)	0.60818 (12)	0.0238 (6)	0.8598 (17)
H14	0.4969	0.2135	0.5940	0.029*	0.8598 (17)
C13	0.4351 (2)	0.3289 (4)	0.66008 (13)	0.0251 (6)	0.8598 (17)
H13	0.4935	0.3215	0.6817	0.030*	0.8598 (17)
C8	0.34672 (17)	0.2150 (4)	0.52053 (11)	0.0221 (6)	0.8598 (17)
H8	0.2831	0.1722	0.5086	0.026*	0.8598 (17)
C7	0.41994 (17)	0.2112 (3)	0.48387 (10)	0.0209 (5)	0.8598 (17)
C15	0.52002 (18)	0.2729 (4)	0.49672 (11)	0.0223 (6)	0.8598 (17)
C1	0.4052 (2)	0.1547 (4)	0.42761 (11)	0.0221 (6)	0.8598 (17)
C2	0.3269 (2)	0.0340 (5)	0.41280 (15)	0.0243 (7)	0.8598 (17)
H2	0.2827	−0.0136	0.4392	0.029*	0.8598 (17)
C3	0.3133 (3)	−0.0165 (6)	0.36020 (18)	0.0295 (7)	0.8598 (17)
H3	0.2595	−0.0976	0.3508	0.035*	0.8598 (17)
C4	0.3776 (2)	0.0501 (5)	0.32082 (12)	0.0316 (8)	0.8598 (17)
H4	0.3685	0.0140	0.2848	0.038*	0.8598 (17)
C5	0.4552 (2)	0.1704 (5)	0.33516 (12)	0.0319 (7)	0.8598 (17)
H5	0.4989	0.2181	0.3086	0.038*	0.8598 (17)
C6	0.46955 (19)	0.2214 (4)	0.38776 (13)	0.0258 (6)	0.8598 (17)
H6	0.5235	0.3024	0.3969	0.031*	0.8598 (17)
N2	0.6003 (4)	0.322 (2)	0.5051 (3)	0.0275 (13)	0.8598 (17)
Cl1'	0.4211 (3)	−0.0021 (6)	0.26712 (16)	0.0355 (12)	0.1402 (17)
C12'	0.3955 (15)	0.065 (3)	0.3320 (6)	0.0258 (7)	0.1402 (17)
C11'	0.3213 (17)	−0.031 (3)	0.3605 (10)	0.0251 (12)	0.1402 (17)
H11'	0.2808	−0.1233	0.3438	0.030*	0.1402 (17)
C10'	0.3084 (15)	0.012 (3)	0.4131 (9)	0.0230 (8)	0.1402 (17)
H10'	0.2584	−0.0516	0.4330	0.028*	0.1402 (17)
C9'	0.3673 (11)	0.148 (2)	0.4380 (6)	0.0211 (6)	0.1402 (17)
C14'	0.4402 (11)	0.240 (2)	0.4083 (7)	0.0238 (6)	0.1402 (17)
H14'	0.4806	0.3321	0.4253	0.029*	0.1402 (17)
C13'	0.4562 (13)	0.202 (2)	0.3551 (8)	0.0251 (6)	0.1402 (17)
H13'	0.5062	0.2656	0.3352	0.030*	0.1402 (17)
C8'	0.3491 (12)	0.192 (2)	0.4942 (5)	0.0221 (6)	0.1402 (17)
H8'	0.2817	0.1744	0.5054	0.026*	0.1402 (17)
C7'	0.4111 (9)	0.251 (2)	0.5321 (5)	0.0209 (5)	0.1402 (17)
C15'	0.5165 (10)	0.281 (3)	0.5210 (7)	0.0223 (6)	0.1402 (17)
C1'	0.3810 (13)	0.294 (3)	0.5875 (6)	0.0221 (6)	0.1402 (17)
C2'	0.2837 (15)	0.357 (5)	0.6003 (10)	0.0243 (7)	0.1402 (17)
H2'	0.2315	0.3515	0.5746	0.029*	0.1402 (17)
C3'	0.265 (3)	0.429 (9)	0.6513 (12)	0.0295 (7)	0.1402 (17)
H3'	0.2066	0.5025	0.6568	0.035*	0.1402 (17)
C4'	0.3293 (16)	0.396 (4)	0.6948 (8)	0.0316 (8)	0.1402 (17)
H4'	0.3103	0.4223	0.7305	0.038*	0.1402 (17)
C5'	0.4215 (14)	0.322 (3)	0.6819 (7)	0.0319 (7)	0.1402 (17)
H5'	0.4684	0.2946	0.7093	0.038*	0.1402 (17)
C6'	0.4461 (14)	0.288 (3)	0.6300 (8)	0.0258 (6)	0.1402 (17)
H6'	0.5140	0.2566	0.6225	0.031*	0.1402 (17)
N2'	0.598 (3)	0.323 (14)	0.514 (3)	0.0275 (13)	0.1402 (17)

### Atomic displacement parameters (Å^2^)

**Table:**

	*U*^11^	*U*^22^	*U*^33^	*U*^12^	*U*^13^	*U*^23^
Cl1	0.0262 (4)	0.0535 (5)	0.0309 (4)	0.0039 (3)	0.0002 (2)	−0.0111 (3)
C12	0.0270 (15)	0.0252 (14)	0.0252 (16)	0.0003 (12)	0.0022 (11)	−0.0039 (13)
C11	0.0152 (15)	0.023 (3)	0.0373 (14)	0.0016 (17)	0.0025 (11)	0.0000 (13)
C10	0.0133 (15)	0.0221 (16)	0.0336 (13)	−0.0006 (15)	−0.0016 (13)	0.0021 (11)
C9	0.0133 (13)	0.0151 (12)	0.0348 (14)	−0.0033 (11)	−0.0009 (10)	0.0039 (11)
C14	0.0145 (13)	0.0231 (14)	0.0339 (15)	0.0026 (10)	0.0024 (13)	0.0000 (13)
C13	0.0168 (12)	0.0252 (14)	0.0335 (16)	−0.0012 (10)	−0.0025 (12)	0.0013 (14)
C8	0.0137 (11)	0.0163 (12)	0.0362 (15)	0.0001 (9)	−0.0004 (12)	0.0033 (12)
C7	0.0130 (11)	0.0159 (11)	0.0336 (12)	0.0010 (9)	−0.0010 (9)	0.0034 (10)
C15	0.0178 (12)	0.0200 (12)	0.0290 (14)	0.0022 (9)	0.0022 (11)	0.0019 (13)
C1	0.0134 (12)	0.0197 (13)	0.0332 (14)	0.0021 (12)	−0.0008 (10)	0.0021 (11)
C2	0.0190 (17)	0.0195 (15)	0.0344 (14)	−0.0013 (12)	0.0006 (13)	0.0025 (11)
C3	0.0250 (16)	0.0279 (17)	0.0355 (15)	−0.0025 (13)	−0.0046 (12)	0.0017 (12)
C4	0.0303 (17)	0.0355 (17)	0.0289 (16)	0.0033 (13)	−0.0026 (12)	0.0013 (13)
C5	0.0269 (14)	0.0346 (17)	0.0343 (16)	0.0020 (13)	0.0021 (13)	0.0059 (13)
C6	0.0173 (13)	0.0228 (14)	0.0372 (17)	−0.0004 (11)	0.0009 (11)	0.0051 (12)
N2	0.0163 (10)	0.0277 (10)	0.039 (4)	0.0005 (8)	0.0000 (12)	0.000 (3)
Cl1'	0.042 (2)	0.036 (2)	0.029 (2)	0.0029 (19)	0.0032 (17)	−0.0018 (17)
C12'	0.0270 (15)	0.0252 (14)	0.0252 (16)	0.0003 (12)	0.0022 (11)	−0.0039 (13)
C11'	0.0152 (15)	0.023 (3)	0.0373 (14)	0.0016 (17)	0.0025 (11)	0.0000 (13)
C10'	0.0133 (15)	0.0221 (16)	0.0336 (13)	−0.0006 (15)	−0.0016 (13)	0.0021 (11)
C9'	0.0133 (13)	0.0151 (12)	0.0348 (14)	−0.0033 (11)	−0.0009 (10)	0.0039 (11)
C14'	0.0145 (13)	0.0231 (14)	0.0339 (15)	0.0026 (10)	0.0024 (13)	0.0000 (13)
C13'	0.0168 (12)	0.0252 (14)	0.0335 (16)	−0.0012 (10)	−0.0025 (12)	0.0013 (14)
C8'	0.0137 (11)	0.0163 (12)	0.0362 (15)	0.0001 (9)	−0.0004 (12)	0.0033 (12)
C7'	0.0130 (11)	0.0159 (11)	0.0336 (12)	0.0010 (9)	−0.0010 (9)	0.0034 (10)
C15'	0.0178 (12)	0.0200 (12)	0.0290 (14)	0.0022 (9)	0.0022 (11)	0.0019 (13)
C1'	0.0134 (12)	0.0197 (13)	0.0332 (14)	0.0021 (12)	−0.0008 (10)	0.0021 (11)
C2'	0.0190 (17)	0.0195 (15)	0.0344 (14)	−0.0013 (12)	0.0006 (13)	0.0025 (11)
C3'	0.0250 (16)	0.0279 (17)	0.0355 (15)	−0.0025 (13)	−0.0046 (12)	0.0017 (12)
C4'	0.0303 (17)	0.0355 (17)	0.0289 (16)	0.0033 (13)	−0.0026 (12)	0.0013 (13)
C5'	0.0269 (14)	0.0346 (17)	0.0343 (16)	0.0020 (13)	0.0021 (13)	0.0059 (13)
C6'	0.0173 (13)	0.0228 (14)	0.0372 (17)	−0.0004 (11)	0.0009 (11)	0.0051 (12)
N2'	0.0163 (10)	0.0277 (10)	0.039 (4)	0.0005 (8)	0.0000 (12)	0.000 (3)

### Geometric parameters (Å, º)

**Table:**

Cl1—C12	1.745 (3)	Cl1'—Cl1'^i^	2.275 (9)
C12—C13	1.389 (4)	C12'—C13'	1.389 (17)
C12—C11	1.397 (5)	C12'—C11'	1.399 (18)
C11—C10	1.371 (4)	C11'—C10'	1.368 (19)
C11—H11	0.9500	C11'—H11'	0.9500
C10—C9	1.398 (4)	C10'—C9'	1.391 (17)
C10—H10	0.9500	C10'—H10'	0.9500
C9—C14	1.407 (4)	C9'—C14'	1.388 (15)
C9—C8	1.462 (4)	C9'—C8'	1.467 (15)
C14—C13	1.382 (4)	C14'—C13'	1.383 (16)
C14—H14	0.9500	C14'—H14'	0.9500
C13—H13	0.9500	C13'—H13'	0.9500
C8—C7	1.344 (3)	C8'—C7'	1.331 (14)
C8—H8	0.9500	C8'—H8'	0.9500
C7—C15	1.442 (3)	C7'—C15'	1.448 (14)
C7—C1	1.483 (4)	C7'—C1'	1.483 (15)
C15—N2	1.146 (5)	C15'—N2'	1.148 (17)
C1—C6	1.402 (4)	C1'—C6'	1.377 (16)
C1—C2	1.402 (4)	C1'—C2'	1.410 (18)
C2—C3	1.382 (5)	C2'—C3'	1.40 (2)
C2—H2	0.9500	C2'—H2'	0.9500
C3—C4	1.393 (5)	C3'—C4'	1.41 (2)
C3—H3	0.9500	C3'—H3'	0.9500
C4—C5	1.390 (4)	C4'—C5'	1.376 (17)
C4—H4	0.9500	C4'—H4'	0.9500
C5—C6	1.384 (4)	C5'—C6'	1.369 (16)
C5—H5	0.9500	C5'—H5'	0.9500
C6—H6	0.9500	C6'—H6'	0.9500
Cl1'—C12'	1.735 (15)		
			
C13—C12—C11	121.7 (3)	C13'—C12'—C11'	122.6 (16)
C13—C12—Cl1	118.7 (2)	C13'—C12'—Cl1'	118.0 (14)
C11—C12—Cl1	119.6 (3)	C11'—C12'—Cl1'	119.1 (15)
C10—C11—C12	118.1 (4)	C10'—C11'—C12'	118 (2)
C10—C11—H11	120.9	C10'—C11'—H11'	120.8
C12—C11—H11	120.9	C12'—C11'—H11'	120.8
C11—C10—C9	122.1 (3)	C11'—C10'—C9'	121.2 (19)
C11—C10—H10	119.0	C11'—C10'—H10'	119.4
C9—C10—H10	119.0	C9'—C10'—H10'	119.4
C10—C9—C14	118.2 (3)	C14'—C9'—C10'	118.6 (14)
C10—C9—C8	117.6 (2)	C14'—C9'—C8'	122.3 (14)
C14—C9—C8	124.2 (2)	C10'—C9'—C8'	119.1 (15)
C13—C14—C9	120.7 (2)	C13'—C14'—C9'	122.5 (15)
C13—C14—H14	119.6	C13'—C14'—H14'	118.8
C9—C14—H14	119.6	C9'—C14'—H14'	118.8
C14—C13—C12	119.0 (2)	C14'—C13'—C12'	116.7 (15)
C14—C13—H13	120.5	C14'—C13'—H13'	121.6
C12—C13—H13	120.5	C12'—C13'—H13'	121.6
C7—C8—C9	129.4 (2)	C7'—C8'—C9'	130.8 (14)
C7—C8—H8	115.3	C7'—C8'—H8'	114.6
C9—C8—H8	115.3	C9'—C8'—H8'	114.6
C8—C7—C15	120.9 (2)	C8'—C7'—C15'	120.8 (13)
C8—C7—C1	124.3 (2)	C8'—C7'—C1'	124.7 (12)
C15—C7—C1	114.7 (2)	C15'—C7'—C1'	114.4 (13)
N2—C15—C7	177.6 (5)	N2'—C15'—C7'	173 (5)
C6—C1—C2	118.2 (3)	C6'—C1'—C2'	114.6 (15)
C6—C1—C7	120.6 (3)	C6'—C1'—C7'	123.4 (15)
C2—C1—C7	121.1 (3)	C2'—C1'—C7'	121.9 (16)
C3—C2—C1	120.7 (3)	C3'—C2'—C1'	119 (2)
C3—C2—H2	119.7	C3'—C2'—H2'	120.4
C1—C2—H2	119.7	C1'—C2'—H2'	120.4
C2—C3—C4	120.8 (4)	C2'—C3'—C4'	123 (2)
C2—C3—H3	119.6	C2'—C3'—H3'	118.6
C4—C3—H3	119.6	C4'—C3'—H3'	118.6
C5—C4—C3	118.9 (3)	C5'—C4'—C3'	115.1 (18)
C5—C4—H4	120.6	C5'—C4'—H4'	122.5
C3—C4—H4	120.6	C3'—C4'—H4'	122.5
C6—C5—C4	120.8 (3)	C6'—C5'—C4'	120.5 (17)
C6—C5—H5	119.6	C6'—C5'—H5'	119.8
C4—C5—H5	119.6	C4'—C5'—H5'	119.8
C5—C6—C1	120.6 (3)	C5'—C6'—C1'	125.6 (17)
C5—C6—H6	119.7	C5'—C6'—H6'	117.2
C1—C6—H6	119.7	C1'—C6'—H6'	117.2
C12'—Cl1'—Cl1'^i^	122.6 (7)		
			
C13—C12—C11—C10	−4.3 (8)	Cl1'^i^—Cl1'—C12'—C11'	−142.8 (8)
Cl1—C12—C11—C10	177.0 (4)	C13'—C12'—C11'—C10'	−0.02 (15)
C12—C11—C10—C9	1.2 (9)	Cl1'—C12'—C11'—C10'	174.0 (14)
C11—C10—C9—C14	2.3 (7)	C12'—C11'—C10'—C9'	0.02 (15)
C11—C10—C9—C8	−179.1 (5)	C11'—C10'—C9'—C14'	−0.1 (3)
C10—C9—C14—C13	−2.9 (4)	C11'—C10'—C9'—C8'	178.7 (14)
C8—C9—C14—C13	178.7 (3)	C10'—C9'—C14'—C13'	0.1 (5)
C9—C14—C13—C12	−0.1 (4)	C8'—C9'—C14'—C13'	−178.6 (15)
C11—C12—C13—C14	3.8 (6)	C9'—C14'—C13'—C12'	−0.1 (4)
Cl1—C12—C13—C14	−177.5 (2)	C11'—C12'—C13'—C14'	0.1 (3)
C10—C9—C8—C7	152.5 (3)	Cl1'—C12'—C13'—C14'	−174.0 (14)
C14—C9—C8—C7	−29.0 (5)	C14'—C9'—C8'—C7'	−31 (3)
C9—C8—C7—C15	−0.2 (4)	C10'—C9'—C8'—C7'	150.5 (17)
C9—C8—C7—C1	−176.9 (2)	C9'—C8'—C7'—C15'	−1 (3)
C8—C7—C1—C6	154.5 (3)	C9'—C8'—C7'—C1'	178.5 (17)
C15—C7—C1—C6	−22.4 (3)	C8'—C7'—C1'—C6'	155 (2)
C8—C7—C1—C2	−25.4 (4)	C15'—C7'—C1'—C6'	−25 (3)
C15—C7—C1—C2	157.7 (3)	C8'—C7'—C1'—C2'	−29 (3)
C6—C1—C2—C3	−0.5 (4)	C15'—C7'—C1'—C2'	151 (2)
C7—C1—C2—C3	179.5 (3)	C6'—C1'—C2'—C3'	8 (5)
C1—C2—C3—C4	0.5 (4)	C7'—C1'—C2'—C3'	−168 (4)
C2—C3—C4—C5	−0.7 (4)	C1'—C2'—C3'—C4'	−18 (8)
C3—C4—C5—C6	0.9 (4)	C2'—C3'—C4'—C5'	13 (7)
C4—C5—C6—C1	−0.9 (4)	C3'—C4'—C5'—C6'	1 (5)
C2—C1—C6—C5	0.6 (4)	C4'—C5'—C6'—C1'	−10 (4)
C7—C1—C6—C5	−179.3 (2)	C2'—C1'—C6'—C5'	5 (4)
Cl1'^i^—Cl1'—C12'—C13'	31.4 (14)	C7'—C1'—C6'—C5'	−178 (2)

Symmetry code: (i) −*x*+1, *y*, −*z*+1/2.

### Hydrogen-bond geometry (Å, º)

*Cg*1 and *Cg*2 are the centroids of rings C1–C6 and C9–C14 of the major disordered component. *Cg*1' and *Cg*2' are the centroids of rings C1'–C6' and C9'–C14' of the minor disordered component.

**Table:**

*D*—H···*A*	*D*—H	H···*A*	*D*···*A*	*D*—H···*A*
C5—H5···Cl1′^i^	0.95	2.69	3.292 (5)	122
C8—H8···N2^ii^	0.95	2.46	3.361 (6)	157
C8—H8···N2′^ii^	0.95	2.53	3.44 (4)	160
C14—H14···N2′	0.95	2.54	3.22 (6)	129
C3—H3···*Cg*1^iii^	0.95	2.99	3.860 (4)	153
C3—H3···*Cg*2′^iii^	0.95	2.95	3.784 (9)	148
C11—H11···*Cg*2^iv^	0.95	2.96	3.418 (7)	111
C11—H11···*Cg*1′^iv^	0.95	2.97	3.486 (15)	115
C14—H14···*Cg*1^v^	0.95	2.81	3.503 (3)	130
C14—H14···*Cg*2′^v^	0.95	2.84	3.585 (8)	136
C3′—H3′···*Cg*2^iv^	0.95	2.59	3.32 (6)	134
C3′—H3′···*Cg*1′^iv^	0.95	2.62	3.39 (6)	139
C6′—H6′···*Cg*1^v^	0.95	2.85	3.52 (2)	129
C6′—H6′···*Cg*2′^v^	0.95	2.93	3.64 (2)	132

Symmetry codes: (i) −*x*+1, *y*, −*z*+1/2; (ii) *x*−1/2, −*y*+1/2, −*z*+1; (iii) −*x*+1/2, *y*−1/2, *z*; (iv) −*x*+1/2, *y*+1/2, *z*; (v) −*x*+1, −*y*, −*z*+1.
